# Digital health promotion strategies to increase HIV testing uptake among key populations: a scoping review

**DOI:** 10.1590/0102-311XEN274825

**Published:** 2026-08-10

**Authors:** Syamikar Baridwan Syamsir, T. Widya Naralia, Hesti Rahayu, Eny Dewi Pamungkas, Raphita Diorarta, Agus Setiawan, Rian Adi Pamungkas, Zainuddin Zainuddin

**Affiliations:** 1 Faculty of Health Sciences, Universitas Pembangunan Nasional Veteran Jakarta, Jakarta, Indonesia.; 2 Faculty of Nursing, Universitas Indonesia, Depok, Indonesia.; 3 Faculty of Health Sciences, Universitas Esa Unggul, Jakarta, Indonesia.; 4 Faculty of Public Health, Universitas Pejuang Republik Indonesia, Makassar, Indonesia.

**Keywords:** HIV Testing, Health Promotion, Digital Technology, Vulnerable Populations, Scoping Review, Teste de HIV, Promoção da Saúde, Tecnologia Digital, Populações Vulneráveis, Revisão de Escopo, Prueba de VIH, Promoción de la Salud, Tecnología Digital, Poblaciones Vulnerables, Revisión de Alcance

## Abstract

HIV testing uptake among key populations remains suboptimal despite its central role in HIV prevention. Digital health promotion strategies have been increasingly implemented to address multilevel barriers to testing. However, current evidence is fragmented across platforms, populations, and settings. This scoping review aimed to map and synthesize current evidence on digital health promotion strategies designed to increase HIV testing uptake among key populations. This scoping review adopted Arksey & O’Malley’s five-stage framework and reported the process following PRISMA-ScR. PubMed, Scopus, the Cochrane Library, and CINAHL were searched for studies published from January 2015 to April 2025. Eligible studies examined digital health promotion strategies focused on HIV testing uptake among key populations. Sixteen studies were included. We identified four thematic clusters of digital health promotion strategies. Overall, the identified approaches showed potential to increase testing uptake and reduce barriers; however, evidence remains heterogeneous and uneven across populations and settings. Digital strategies for HIV testing are complementary. Integrating mobile apps, social media outreach, digital messaging, and peer-driven networks may offer scalable, context-sensitive solutions for key populations. However, the limited representation of certain groups, particularly transgender women and people who inject drugs, highlights important gaps in the evidence base. Further studies with more robust design, wider geographic coverage, and longer follow-up are warranted.

## Resumo

A adoção dos testes de HIV entre populações-chave continua abaixo do ideal, apesar de seu papel central na prevenção do HIV. Estratégias digitais de promoção da saúde têm sido implementadas com mais frequência para enfrentar barreiras multinível aos testes. No entanto, a evidência acerca desse fato se encontra dividida entre plataformas, populações e ambientes. Esta revisão de escopo buscou mapear e sintetizar a evidência acerca de estratégias para promover saúde digital que foram projetadas para aumentar a aceitação de testes de HIV entre populações-chave. Adotou-se o quadro de cinco etapas de Arksey e O’Malley, relatando o processo de acordo com o PRISMA-ScR. Pesquisa bibliográfica foi conduzida nas plataformas PubMed, Scopus, Biblioteca Cochrane e CINAHL buscando estudos publicados entre janeiro de 2015 e abril de 2025. Estudos elegíveis analisaram estratégias de promoção da saúde digital que focaram na aceitação de testes de HIV em populações-chave. Ao todo, 16 estudos foram incluídos. Identificamos quatro grupos temáticos de estratégias de promoção da saúde digital. No geral, as abordagens identificadas podem aumentar a adesão aos testes e reduzir barreiras, a despeito da evidência heterogênea e desigual entre populações e ambientes. Estratégias digitais para testes de HIV são complementares. Integrar aplicativos móveis, alcance em redes sociais, mensagens digitais e redes baseadas em pares pode oferecer soluções escaláveis e sensíveis ao contexto das populações-chave. No entanto, a representação limitada de certos grupos, especialmente mulheres transgênero e pessoas que usam drogas injetáveis, destaca lacunas importantes na base de evidências. Estudos adicionais com desenhos mais robustos, maior cobertura geográfica e acompanhamento mais longo são necessários.

## Resumen

La adopción de pruebas de VIH entre las poblaciones clave sigue estando por debajo de lo ideal, a pesar de su papel fundamental para prevenir el VIH. Las estrategias digitales de promoción de la salud se han implementado cada vez más para hacer frente a las barreras multinivel para la realización de pruebas. Sin embargo, la evidencia sobre este hecho está dividida entre plataformas, poblaciones y entornos. Esta revisión de alcance tuvo como objetivo mapear y sintetizar la evidencia sobre estrategias para promover la salud digital, diseñadas para aumentar la aceptación de las pruebas de VIH entre poblaciones clave. Se adoptó la metodología de cinco etapas de Arksey & O’Malley, reportando el proceso según el PRISMA-ScR. La investigación bibliográfica se realizó en las plataformas PubMed, Scopus, Biblioteca Cochrane y CINAHL mediante la búsqueda de estudios publicados entre enero de 2015 y abril de 2025. Los estudios elegibles analizaron las estrategias de promoción de la salud digital, centradas en la aceptación de pruebas de VIH en poblaciones clave. En total, se incluyeron 16 estudios. Identificamos cuatro grupos temáticos de estrategias de promoción de la salud digital. En general, los enfoques identificados pueden aumentar la adherencia a las pruebas y reducir las barreras, a pesar de la evidencia heterogénea y desigual entre poblaciones y entornos. Las estrategias digitales para pruebas de VIH son complementarias. Integrar las aplicaciones móviles, el alcance en redes sociales, los mensajes digitales y las redes basadas en pares puede ofrecer soluciones escalables y sensibles al contexto de las poblaciones clave. Sin embargo, la limitada representación de determinados grupos, sobre todo las mujeres transgénero y las personas que consumen drogas inyectables, resalta importantes brechas en la base de evidencias. Se necesitan más estudios con diseños más robustos, mayor cobertura geográfica y seguimiento más prolongado.

## Introduction

HIV/AIDS remains a major global public health concern. In 2024, an estimated 40.8 million people were living with HIV, with approximately 630,000 deaths reported worldwide, 65% of cases concentrated in the WHO (World Health Organization) African Region, and an estimated 5.3 million people unaware of their HIV status ^1^. Delayed diagnosis contributes to increased mortality and continued HIV transmission ^2^. HIV testing plays a central role in epidemic control because it enables timely initiation of antiretroviral therapy (ART) and guides HIV-negative individuals as to prevention options such as pre-exposure prophylaxis (PrEP), both of which improve outcomes and reduce onward transmission ^3^. However, global testing coverage remains suboptimal due to factors such as stigma, discrimination, limited service availability, and concerns about confidentiality ^4^. In addition, substantial regional disparities persist, with variations in HIV incidence and testing coverage across regions. While sub-Saharan Africa bears the highest burden, other regions such as Latin America and Eastern Europe continue to report considerable numbers of new infections alongside uneven progress in testing and treatment coverage ^5^. When individuals are unaware of their HIV status, early diagnosis opportunities are missed, disease progression often goes undetected, and health care costs rise, with economic modelling showing that early access to care is cost-saving and prevents secondary infections ^6^. Limited testing reinforces social stigma and psychosocial stress, particularly in settings with low health literacy ^7^ ^,^ ^8^.

Testing inequalities are most evident among key populations, including men who have sex with men (MSM), transgender people, sex workers, and people who inject drugs (PWID), whose HIV prevalence is substantially higher than in the general population ^9^. Structural factors such as criminalization, discrimination, and the scarcity of humanized and inclusive healthcare services further heighten the vulnerability of these groups ^10^. Strategies to increase HIV testing coverage should therefore prioritize these populations through contextual, participatory, and needs-oriented approaches. Understanding the drivers and barriers of testing decisions is essential. Common facilitators include perceived personal risk, social support, ease of access to services, and the presence of HIV-related symptoms ^11^. Barriers include stigma and discrimination, fear of a positive result, limited HIV knowledge, and concerns about confidentiality ^11^ ^,^ ^12^. These insights can underpin more responsive interventions such as targeted counselling and enhanced social support during and after HIV self-testing distribution ^13^. These structural barriers underscore the need for innovative and context-sensitive approaches, with digital health strategies offering potential to improve confidentiality, expand access, and address limitations of conventional HIV testing services.

Advances in digital technology create new opportunities to expand HIV testing. Telehealth, mobile health applications, social media campaigns, and SMS-based reminder systems have been shown to extend service reach, reduce distance-related barriers, and improve access to HIV-related information and testing services, particularly in resource-constrained settings ^14^ ^,^ ^15^. Digital strategies enable more personalized health promotion through tailored support and promote interaction through social features and peer support, which helps deliver relevant and accessible health messages to users ^16^. Other evidence indicates that digital media can increase engagement with and acceptability of health interventions, particularly among vulnerable populations ^17^ ^,^ ^18^.

Despite the increase in digital health promotion strategies, there is still a gap in the systematic documentation and mapping of these approaches, especially for key populations across diverse settings. Previous scoping reviews have discussed digital HIV testing promotion strategies, including social network-based approaches ^19^. However, their scope was largely confined to social network-based methods. To date, no scoping review has comprehensively mapped the full spectrum of digital health promotion strategies to increase HIV testing uptake among key populations across global contexts. Therefore, this scoping review aims to identify and map digital technology-based health promotion strategies used to expand HIV testing among key populations. The findings are intended to provide a conceptual and empirical basis for developing more effective digital interventions, inform evidence-based health policy, and reinforce global efforts to control the HIV epidemic.

## Methods

### Design

This scoping review adopted Arksey & O’Malley’s five-stage methodological framework, comprising: (1) identifying the research question, (2) identifying relevant studies, (3) selecting studies, (4) charting the data, and (5) collating, summarizing, and reporting the results ^20^. The reporting followed the *Preferred Reporting Items for Systematic Reviews and Meta-Analyses extension for Scoping Reviews* (PRISMA-ScR) checklist ^21^.

#### Stage 1: Identifying the research question

This review was guided by the following research question: what digital health promotion strategies have been implemented to increase HIV testing uptake among key populations? The question was formulated to capture a broad range of digital health interventions that aim to increase HIV testing uptake specifically within key populations, including MSM, transgender people, sex workers, PWID, and adolescents and young people within key populations. This formulation aligns with the exploratory purpose of a scoping review, which is to map existing evidence and identify knowledge gaps rather than evaluate intervention effectiveness. Given the heterogeneity of digital health interventions, study designs, and implementation contexts, as well as the fragmented nature of the available evidence, a scoping review approach was considered more appropriate than a systematic review. This approach enables comprehensive mapping of diverse strategies and identification of research gaps, rather than focusing on the effectiveness of specific interventions ^22^.

#### Stage 2: Identifying relevant studies

The identification of relevant studies was guided by the Population-Concept-Context (PCC) framework as recommended by the JBI for scoping reviews ^23^. The population included individuals from key populations, such as MSM, transgender people, sex workers, PWID, and adolescents and young people within key populations. Studies involving general populations were included only if they explicitly addressed these subgroups. The concept focused on health promotion strategies using digital technologies designed to increase HIV testing uptake, including HIV self-testing approaches. The context encompassed all geographical and sociocultural settings globally, without restrictions on location or healthcare system characteristics.

This scoping review considered a broad range of study designs, including randomized controlled trials (RCTs), quasi-experimental studies, observational studies, mixed-methods studies, and study protocols, to comprehensively map current evidence on digital health promotion strategies for HIV testing uptake. This approach is consistent with the purpose of scoping reviews, which aim to address broad research questions and include heterogeneous evidence sources ^22^.

A systematic search was conducted in four electronic databases: PubMed, Scopus, the Cochrane Library, and CINAHL. These databases were selected to capture a comprehensive body of biomedical evidence (PubMed), multidisciplinary and international literature (Scopus), evidence syntheses (Cochrane Library), and nursing, behavioral, and community-based studies relevant to digital health interventions (CINAHL).

The search strategy combined keywords, Boolean operators (AND, OR), and *Medical Subject Headings* (MeSH) where applicable. Search terms were organized into four conceptual domains: digital health (including mobile, web-based, and social media-related approaches), HIV, health promotion, and key populations. These domains were combined using the AND operator to retrieve studies aligned with the scope of this review.

The search strategy was developed iteratively to ensure both sensitivity and specificity, with refinement of keywords and combinations across databases. The initial strategy was developed for PubMed and subsequently adapted for the other databases, taking into account differences in indexing systems, thesaurus structures, and controlled vocabularies used in each database. To enhance transparency and reproducibility, full search strategies for each database, including Boolean logic, nested keyword combinations, and MeSH term applications, are provided in Supplementary Material (https://cadernos.ensp.fiocruz.br/static//arquivo/suppl-e00274825_8421.pdf).

#### Stage 3: Selecting studies

All records identified through the database searches were imported into Mendeley Reference Manager (https://www.mendeley.com) for duplicate removal. Study selection was conducted in two stages: an initial screening of titles and abstracts, followed by full-text assessment. Two independent reviewers applied predefined inclusion and exclusion criteria at both stages. Articles that were considered potentially relevant or unclear during the initial screening were retained for full-text review. Any discrepancies between reviewers were resolved through discussion until consensus was reached.

Studies were included if they focused on digital health promotion strategies aimed at increasing HIV testing uptake among key populations, including MSM, transgender people, sex workers, PWID, and adolescents and young people within key populations. Eligible studies were published in English between January 2015 and April 2025. Studies were excluded if they did not involve digital health promotion strategies, did not report outcomes related to HIV testing uptake, or were conducted in general populations without specific analysis of key populations. Studies reporting indirect, behavioral, feasibility, or implementation-related outcomes relevant to HIV testing uptake were retained if they provided meaningful insights into digital HIV testing promotion strategies. Non-empirical publications, such as editorials, commentaries, and opinion pieces, were also excluded.

Study protocols and modelling studies were included when they provided detailed descriptions of planned or simulated digital health promotion strategies relevant to HIV testing uptake. These studies were used to map intervention components but were not considered as empirical outcome evidence. Screening decisions were documented using a structured spreadsheet in Google Sheets (https://docs.google.com/spreadsheets). The restriction to English-language publications was applied to ensure consistency in data extraction and interpretation, while recognizing the potential exclusion of relevant studies from non-English-speaking and low-resource settings. The final search was conducted in April 2025.

**Figure 1:**
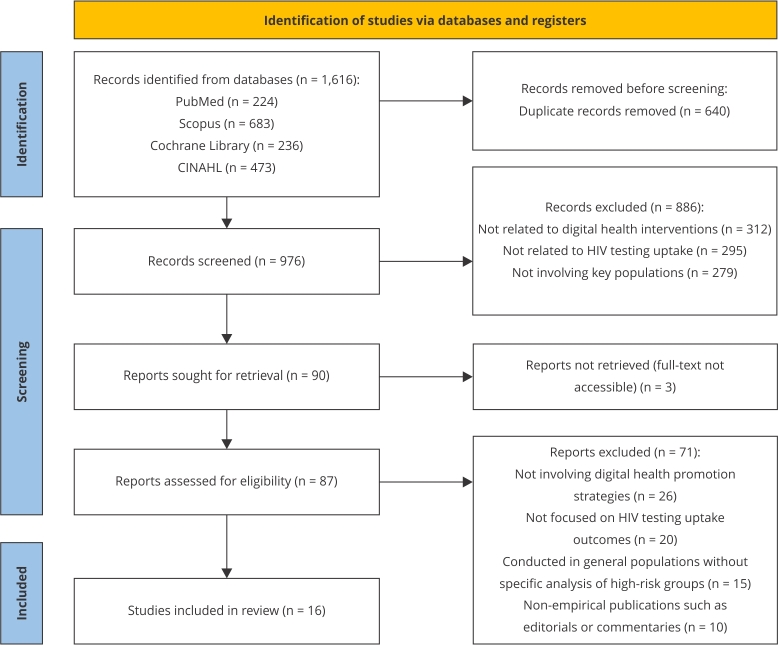
PRISMA (*Preferred Reporting Items for Systematic Reviews and Meta-Analyses*) 2020 flow diagram of the study selection process.

The study selection process was documented and reported following the PRISMA-ScR guidelines. A PRISMA 2020 flow diagram (Figure 1) presents the number of records identified, screened, assessed for eligibility, and included in the final review.

#### Stage 4: Charting the data

Data extraction adopted a standardized charting form developed for this review. The data extraction form used in this review is provided in Supplementary Material (https://cadernos.ensp.fiocruz.br/static//arquivo/suppl-e00274825_8421.pdf). The form captured key study characteristics, including: author(s) and year of publication, country or region, study population (restricted to key populations such as MSM, transgender people, sex workers, PWID, and adolescents and young people within key populations), sample size, study design, study objectives, type of digital health promotion strategy or intervention, key findings related to HIV testing uptake, and thematic category or subtheme.

Two reviewers independently piloted the charting form on six randomly selected articles to ensure clarity, consistency, and feasibility ^24^. Based on this pilot, the charting form was refined to ensure systematic and comprehensive capture of all relevant information.

To enhance transparency, the methodological quality of included studies was appraised but not used as an exclusion criterion. Quantitative, qualitative, and mixed-methods studies were assessed using the *Mixed Methods Appraisal Tool* (MMAT, 2018 version) ^25^, while review studies (systematic or scoping) were appraised using the *Joanna Briggs Institute Critical Appraisal Checklist for Systematic Reviews and Research Syntheses* ^26^. Appraisal results were summarized descriptively to provide context regarding the strengths and limitations of the available evidence. These assessments informed interpretation of the findings without being used as exclusion criteria. Two reviewers conducted the appraisal independently, with any disagreements resolved through discussion.

#### Stage 5: Collating, summarizing, and reporting the results

The included studies were grouped by article type, study design, country or region, key population, and the type of digital health promotion strategy implemented. A descriptive numerical summary of study characteristics was first generated to provide an overview of the included evidence.

Thematic analysis was then conducted using an iterative and inductive approach, consistent with established guidance for scoping reviews ^24^. Two reviewers independently coded the extracted data to identify initial concepts related to digital health promotion strategies for HIV testing uptake. These codes were subsequently organized into subthemes, which were further refined and consolidated into broader thematic categories through discussion and consensus. Discrepancies in coding and theme development were resolved through iterative comparison and agreement between reviewers. Data coding and management were conducted manually using structured spreadsheets. This process ensured consistency and transparency in the development of themes and subthemes. The predefined thematic categories included mobile app-based interventions, social media and web-based outreach, messaging-based strategies, and peer- and network-based digital approaches.

Finally, the identified themes were compared across studies to identify overarching patterns and variations in digital health promotion strategies. A narrative thematic synthesis of the findings is presented in the *Results* section.

## Results

### Study selection

The initial search across four electronic databases (Scopus, PubMed, the Cochrane Library, and CINAHL) retrieved 1,616 records. After deduplication and sequential screening of titles, abstracts, and full texts, 16 studies met the inclusion criteria and were included in this scoping review. During the title and abstract screening stage, studies were excluded primarily because they were not related to digital health interventions, did not address HIV testing uptake, or did not involve key populations. Studies were excluded at the full-text reading stage primarily because they did not involve digital health promotion strategies, did not focus on HIV testing uptake outcomes, or targeted general populations without addressing key populations. In line with the protocol, study protocols and modelling studies were retained when they provided substantial descriptions of planned or simulated digital strategies relevant to HIV testing uptake in key populations, although they did not contribute empirical outcome data. The selection process is summarized in the PRISMA 2020 flow diagram (Figure 1).

### Characteristics of included studies

A total of 16 studies were included, with most conducted in the United States and Asian settings, particularly China and Hong Kong, alongside several studies from Malaysia, India, and broader low- and middle-income countries (LMICs) or multi-country contexts. Most studies focused on MSM or young MSM, while a smaller number addressed transgender people, sex workers, adolescents, or other key populations. Sample sizes varied widely, ranging from small pilot trials with fewer than 50 participants to large-scale RCTs, program evaluations, and review-level evidence involving several thousand participants across multiple settings.

Across the included studies, digital interventions were implemented in diverse contexts, including community-based organizations (CBOs), public health programs, and digitally mediated environments such as mobile applications and online platforms. Several interventions were delivered through community-based organizations or outreach programs ^27^ ^,^ ^28^, while others operated within broader digital ecosystems, including app-based services and online advertising platforms ^29^ ^,^ ^30^ ^,^ ^31^ ^,^ ^32^. These variations reflect differences in implementation settings, with most studies evaluating active digital interventions targeting MSM and young MSM populations, and highlight the potential scalability of digital interventions across community and digitally integrated health systems.

With respect to study design, the included studies comprised RCTs (n = 3) plus one pilot RCT (n = 1), one quasi-experimental study (n = 1), mixed-methods feasibility or pilot studies (n = 3), cross-sectional quantitative studies (n = 2), one quantitative program case study (n = 1), systematic reviews and meta-analyses (n = 2), and study protocols (n = 3). Pilot RCTs and protocols were preserved as distinct categories to avoid inflating efficacy evidence, as these studies primarily describe planned or preliminary interventions rather than providing empirical outcome data. This heterogeneity reflects the evolving state of research on digital strategies for HIV testing, in which both formative pilot work and large-scale evaluations contribute to the evidence base.

Across the studies, mobile applications (approximately six studies) emerged as the most frequently reported digital approach. Common features included personalized reminders, geolocation services, HIV self-testing (HIVST) kit ordering, and linkage to information about PrEP. Social media campaigns and SMS-based reminders were also widely applied, particularly in settings with broad digital access. Several studies leveraged peer-network strategies, including peer recruitment and secondary distribution of HIVST kits through digital platforms, as well as crowdsourced interventions. Two systematic reviews and meta-analyses synthesized evidence across multiple contexts and reported pooled associations between digital strategies and higher HIV testing uptake among key populations. Overall, multiple studies documented higher HIV testing uptake associated with digital strategies, with effects most consistently observed in app-based and social-media-based interventions among MSM and young MSM, while other key populations remained less represented in the evidence base. Detailed study characteristics are summarized in Box 1.

**Box 1:** Characteristics of included studies (n = 16).

No.	STUDY (YEAR)	COUNTRY/REGION	POPULATION	SAMPLE SIZE	STUDY DESIGN	OBJECTIVE	DIGITAL STRATEGY/ INTERVENTION	KEY FINDINGS (HIV TESTING UPTAKE)
1	He et al. ^38^ (2025)	China (Zhejiang Province)	MSM	n = 4,600	Cross-sectional (quantitative descriptive)	Compare the effectiveness of mobile social media-based vs. volunteer-driven recruitment	Mobile social media vs. volunteer-driven recruitment; mailed HIVST vs. offline testing	Mobile social media recruited more first-time testers (19.5% vs. 17.4%); higher positivity (1.7% vs. 0.9%); associated with mail-in HIVST (aOR = 2.02)
2	Biello et al. ^33^ (2019)	United States (Boston and Bronx)	Young MSM (15-24 years)	Phase 1: n ≈ 30; phase 2: n ≈ 15; pilot RCT: n = 60	Protocol - pilot RCT (3-phase)	Describe the development and initial testing of the MyChoices app	MyChoices mobile app: self-assessment, reminders, geofencing, HIVST kit ordering, PrEP information	No empirical results (protocol); expected to increase HIV testing and PrEP uptake
3	Wang et al. ^28^ (2024)	Hong Kong SAR	MSM (Chinese-speaking)	Intervention: n = 400; control: n = 412	Quasi-experimental (prospective comparison, 6-month follow-up)	Evaluate an online HIVST promotion program run by a CBO during the pandemic	Online video; webpage information; HIVST kit delivery; live chat; SMS/instant messaging reminders	Greater HIV testing uptake (57% vs. 49%; aOR = 1.43); higher HIVST uptake (25.8% vs. 14.8%; aOR: 2.04)
4	Nicely et al. ^39^ (2025)	LMICs (Kenya, South Africa, Uganda, China)	MSM, refugee youth, CBO users	5 studies; total n = 2,581	Systematic review and meta-analysis	Assess digital health modalities in LMICs	SMS, social media, HIVST apps, online peer groups	Pooled HIVST uptake 83.5% vs. 66.8%; confirmation 82.5% vs. 25.1%
5	Shrestha et al. ^36^ (2023)	Malaysia (Kuala Lumpur)	MSM (PrEP-naive, HIV-negative or unknown status)	n = 50	Mixed-methods (single-arm beta test, 30-day follow-up)	Evaluate the JomPrEP mobile app	JomPrEP app: HIVST ordering, e-consultation, reminders, rewards	84% ordered an HIVST kit; 95% verified results; the app facilitated access to testing
6	Liu et al. ^34^ (2019)	United States (Chicago and Tampa)	Young MSM (15-24 years)	Pilot RCT: n = 60 (2:1)	Protocol - pilot RCT	Develop the LYNX app	LYNX app: sexual diary, personalized risk score, reminders, HIVST ordering, geolocation, PrEP information	No empirical results (protocol)
7	Biello et al. ^29^ (2021)	United States (Boston and Bronx)	Young MSM (15-24 years)	Theatre testing: n = 28; technology pilot: n = 11	Mixed-methods (formative plus pilot)	Develop and evaluate the MyChoices app	MyChoices app: reminders, GPS locator, HIVST kit ordering, safer-sex supplies, PEP information	91% used the app; 91% felt motivated to test for HIV; 73% reported the app facilitated testing
8	Lin et al. ^40^ (2023)	China (Shandong, 11 cities)	MSM (≥ 18 years)	Enrolled: n = 935; intervention: n = 404; control: n = 531	Cluster RCT (12-month follow-up)	Evaluate a multilevel crowdsourced intervention via WeChat	WeChat digital materials; free HIVST kits; peer groups; videos	Higher HIV testing at 6-12 months; peer groups and video components were most effective
9	Biello et al. ^31^ (2025)	United States (9 cities)	Sexual minority men (15-29 years)	n = 381 (RCT, 3-arm)	RCT (6-month follow-up)	Compare MyChoices and LYNX vs. standard of care	Apps: reminders, HIVST kit ordering, geolocation, PrEP information	HIV testing at least once over 6 months: 74% (MyChoices) vs. 59% (standard of care), RR = 1.27
10	Biello et al. ^30^ (2022)	United States (Boston, Bronx, Chapel Hill)	Young MSM (15-24 years)	n = 60 (2:1 RCT)	Pilot RCT (6-month follow-up)	Evaluate preliminary efficacy of the MyChoices app	MyChoices: reminders, geolocation, HIVST ordering, multimedia content	HIV testing uptake: 79% vs. 65% (not statistically significant); acceptable usability (SUS, mean = 75.8)
11	Lightfoot et al. ^27^ (2018)	United States (California)	African-American and Latino MSM (18-45 years)	Peer recruiters: n = 28; HIVST kits: n = 165	Quantitative descriptive (network evaluation)	Evaluate HIVST distribution through peer recruiters	Peer recruiters distributed HIVST via CBOs, venues, and apps	Higher positivity 6.14% vs. 1.49%; reached non-testers
12	Veronese et al. ^41^ (2020)	Global (HIC and LMIC: United States, Hong Kong, Taiwan, China, Peru, India)	MSM and TGW	13 studies; total: n = 8,875	Systematic review and meta-analysis	Assess the impact of digital communication technologies on HIV testing	Social media, online video, instant messaging/email, live chat, HIVST referral	Pooled RR for testing uptake = 1.5; social media RR = 1.7
13	Zhou et al. ^37^ (2022)	China (68 cities)	MSM (≥ 18 years)	Index: n = 309; control: n = 102; alters: n = 334	RCT (3-arm, online)	Evaluate peer referral with cash incentives	WeChat kit ordering and reporting; referral links; e-incentives	Increased number of testers (IRR: 2.98 to 3.26); increased newly tested alters (IRR: 3.49 to 4.22)
14	Chaudary et al. ^35^ (2024)	India (Mumbai-Thane)	MSM (≥ 18 years, non/recent testers)	Target: n = 1,000	Protocol - RCT (3-arm)	Test the effectiveness of CHALO! 2.0	WhatsApp multimedia campaign; digital coupons; peer outreach	Protocol; results not yet available
15	Lee et al. ^42^ (2022)	United States (Washington State)	Latinx immigrant sexual minority men (≥ 18 years)	Focus groups: n = 15; website visitors: n = 739; survey: n = 60	Mixed-methods (feasibility)	Evaluate a culturally tailored social media campaign	Social media (Facebook/Instagram) leading to a bilingual website with HIV testing/PrEP information	739 users reached; 70% had tested in the past 6 months; effectiveness for uptake not measured
16	Chambers et al. ^32^ (2024)	United States (Rhode Island)	MSM (gay and bisexual)	2,536,405 impressions; 35,022 clicks; 225 conversions	Quantitative descriptive (case study)	Evaluate performance of a multi-platform digital advertising programme	Google Search, Display, Grindr, and Facebook ads	Google Search click-through rate 7.1%; conversion rate 7.0% (proxy for uptake)

aOR: adjusted odds ratios; CBO: community-based organization; GPS: global positioning system; HIC: high-income countries; HIVST: HIV self-testing; IRR: incidence rate ratio; LMIC: low- or middle-income countries; MSM: men who have sex with men; PEP: post-exposure prophylaxis; PrEP: pre-exposure prophylaxis; RCT: randomized controlled trial; RR: risk ratio; SUS: *System Usability Scale*; TGW: transgender women.

### Methodological quality appraisal

Although quality appraisal is not mandatory in scoping reviews, a descriptive assessment was conducted to contextualize the robustness of the evidence without serving as an exclusion criterion. Among the 11 primary studies appraised, overall methodological quality was moderate to high, with no study scoring below 3 out of 5 MMAT criteria. Three study protocols ^33^ ^,^ ^34^ ^,^ ^35^ were also descriptively appraised to document planned methodological rigor and were not considered outcome evidence. The two included systematic reviews achieved high methodological quality ratings (10/11), indicating robust methodological rigor despite minor limitations. Detailed appraisal results are presented in Boxes 2 and 3.

**Box 2:** Quality appraisal of primary studies using *Mixed Methods Appraisal Tool* (MMAT, 2018 version).

No. *	STUDY (YEAR)	STUDY DESIGN	Q1	Q2	Q3	Q4	Q5	OVERALL QUALITY
1	He et al. ^38^ (2025)	Quantitative descriptive	Yes	No	Yes	No	Yes	3/5 (moderate)
2	Biello et al. ^33^ (2019)	Protocol (pilot RCT)	Yes	Yes	No	No	Yes	3/5 (moderate - protocol)
3	Wang et al. ^28^ (2024)	Quantitative non-randomized	No	Yes	Yes	Yes	Yes	4/5 (high)
5	Shrestha et al. ^36^ (2023)	Mixed methods	Yes	Yes	Yes	Yes	No	4/5 (high)
6	Liu et al. ^34^ (2019)	Protocol (pilot RCT)	Yes	Yes	No	No	Yes	3/5 (moderate - protocol)
7	Biello et al. ^29^ (2021)	Mixed methods	Yes	Yes	Yes	Yes	No	4/5 (high)
8	Lin et al. ^40^ (2023)	RCT, cluster	Yes	Yes	Yes	No	Yes	4/5 (high)
9	Biello et al. ^31^ (2025)	RCT (3-arm)	Yes	Yes	Yes	No	Yes	4/5 (high)
10	Biello et al. ^30^ (2022)	Pilot RCT	Yes	Yes	Yes	No	Yes	4/5 (high)
11	Lightfoot et al. ^27^ (2018)	Quantitative descriptive	Yes	No	Yes	No	Yes	3/5 (moderate)
13	Zhou et al. ^37^ (2022)	RCT	Yes	Yes	Yes	No	Yes	4/5 (high)
14	Chaudary et al. ^35^ (2024)	Protocol (RCT)	Yes	Yes	No	Yes	Yes	4/5 (high - protocol)
15	Lee et al. ^42^ (2022)	Mixed methods	Yes	Yes	Yes	No	No	3/5 (moderate)
16	Chambers et al. ^32^ (2024)	Quantitative descriptive	Yes	No	Yes	No	Yes	3/5 (moderate)

RCT: randomized controlled trial.

Note: Q1 - study design appropriateness; Q2 - data quality; Q3 - outcome completeness; Q4 - risk of bias; Q5 - analytical rigor, adapted from the MMAT.

* Numbering aligns with Box 1.

**Box 3:** Quality appraisal of systematic reviews using the JBI 11-item checklist.

No. *	STUDY (YEAR)	STUDY DESIGN	Q1	Q2	Q3	Q4	Q5	Q6	Q7	Q8	Q9	Q10	Q11	OVERALL QUALITY
4	Nicely et al. ^39^ (2025)	Systematic review & meta-analysis	Yes	Yes	Yes	Yes	Yes	Yes	Yes	Yes	No	Yes	Yes	10/11 (High)
12	Veronese et al. ^41^ (2020)	Systematic review & meta-analysis	Yes	Yes	Yes	Yes	Yes	Yes	Yes	Yes	Yes	Yes	No	10/11 (High)

Note: Q1 - review question clarity; Q2 - appropriateness of inclusion criteria; Q3 - adequacy of search strategy; Q4 - adequacy of sources; Q5 - appropriateness of appraisal criteria; Q6 - independent critical appraisal; Q7 - error minimization in data extraction; Q8 - appropriateness of synthesis methods; Q9 - assessment of publication bias; Q10 - support for recommendations; Q11 - appropriateness of future research directions (JBI critical appraisal checklist).

* Numbering aligns with Box 1.

### Thematic analysis

A thematic analysis was conducted to map the range of digital, technology-enabled health promotion strategies used to increase HIV testing uptake among key populations. From the 16 included studies, four overarching themes emerged that represent consistent intervention patterns across studies. These themes reflect diverse technological approaches, spanning mobile applications, social media and web-based outreach, digital messaging, and peer- and network-based strategies. A summary of themes, subthemes, and operational definitions is presented in Box 4.

**Box 4:** Thematic analysis.

THEME	SUBTHEME	DEFINITION
Theme 1: Mobile app-based strategies	Mobile apps	Purpose-built mobile applications that support HIV testing and prevention, functioning as integrated platforms for digital services, for example MyChoices, LYNX, and JomPrEP
	Personalized reminders	Tailored notifications based on user profiles or needs to prompt routine and scheduled HIV testing
	Gamification and rewards	Game elements, points, or incentives embedded in apps to enhance motivation to test for HIV
	Integration with clinic and e-consultation	App-based links to clinic services, including e-consultation, chat with health professionals, and access to results or test ordering
	Geolocation and in-app support	Location-based features and in-app support such as chat or real-time information to facilitate access to HIV testing services
	Digital HIVST ordering and reporting (app)	In-app facilities for ordering HIVST kits and reporting results digitally
Theme 2: Social media and web-based outreach	Social media campaigns	Educational and promotional HIV testing campaigns delivered through social media platforms, using general or targeted messaging
	Online video campaigns	Dissemination of educational video or multimedia content online to increase motivation for HIV testing
	Culturally tailored social media	Social media campaigns adapted to the culture, language, or context of target communities
	Website click-through	Strategies that direct users from social media or advertisements to dedicated landing pages for information or access to HIV testing services
	Web-based education	Provision of structured, interactive web pages with HIV testing information and guidance
	Targeted digital advertising	Paid digital advertisements on search engines, display networks, or geosocial apps targeting at-risk populations to increase testing uptake
	Digital HIVST facilitation (web or social)	Web or social media-based facilitation of HIV self-testing, including kit ordering, instructions, live-chat assistance, and result reporting
Theme 3: Digital messaging (SMS and WhatsApp)	SMS campaigns	Short message service reminders, education, or promotional messages to increase HIV testing uptake
	WhatsApp campaigns	Educational broadcasts or two-way interactions via WhatsApp or WhatsApp Business to support HIV testing decisions
Theme 4: Peer and network-based digital strategies	Peer distribution of HIVST	Distribution of HIVST kits through peer networks supported by digital tools to expand reach
	Digital peer recruitment	Recruitment for HIV testing through digital platforms leveraging social or peer networks
	Peer referral links	Unique digital links that enable peers to refer others to order HIVST kits
	Incentive-based strategies	Monetary or digital coupon incentives to encourage peer referral or participation in HIV testing
	Peer-moderated online groups	Online groups moderated by peers to provide social support and encourage HIV testing uptake

HIVST: HIV self-testing.

### Theme 1: Mobile app-based strategies

Mobile applications have emerged as a core digital strategy to support HIV testing, particularly among young MSM and MSM. Apps such as MyChoices, LYNX, and JomPrEP function as integrated platforms that enable users to plan testing, receive personalized reminders, access HIV self-testing kits, and utilize geolocation features to identify nearby services, thereby facilitating timely testing and linkage to care ^29^ ^,^ ^30^ ^,^ ^31^ ^,^ ^33^ ^,^ ^34^ ^,^ ^36^ ^,^ ^37^. Randomized and pilot studies indicate that in-app personalization can improve adherence to recommended testing schedules, while gamification elements enhance motivation to test ^30^ ^,^ ^31^ ^,^ ^34^ ^,^ ^36^. Clinical integration further extends functionality through e-consultation and access to results, which improves linkage to care ^36^. In addition, app-based ordering of HIVST kits, digital result reporting, and pre- or post-test counselling position the app as a bridge between individuals and formal health services ^29^ ^,^ ^30^ ^,^ ^31^ ^,^ ^33^ ^,^ ^34^ ^,^ ^36^ ^,^ ^37^. Considered in conjunction, mobile apps operate not only as promotional tools but also as integrated platforms that facilitate testing uptake by enabling access to HIV self-testing, providing personalized reminders, and improving linkage to care among key populations.

### Theme 2: Social media and web-based outreach

Beyond mobile apps, social media and web platforms play a substantial role in engaging communities with HIV testing. Campaigns on platforms such as WeChat, Facebook, and Instagram have been effective in reaching key populations, as reflected in increased HIV testing uptake, engagement metrics such as click-through rates, and linkage to testing services ^28^ ^,^ ^38^ ^,^ ^39^ ^,^ ^40^ ^,^ ^41^. These approaches are reinforced by interactive multimedia content, including educational videos that can increase motivation to test ^28^ ^,^ ^29^ ^,^ ^41^, as well as culturally tailored content that aligns with the language and norms of target communities ^42^. Click-through mechanisms route users from social media or digital adverts to dedicated landing pages where they can obtain information or order HIVST kits ^28^ ^,^ ^32^ ^,^ ^42^. Paid digital advertising expands audience reach, while web-based facilitation supports kit distribution, instructions for use, and follow-up assistance ^28^ ^,^ ^35^ ^,^ ^38^ ^,^ ^39^ ^,^ ^40^ ^,^ ^41^. In combination, social media and the web provide channels that convert information into concrete actions, contributing meaningfully to increasing HIV testing uptake.

### Theme 3: Digital messaging (SMS and WhatsApp)

Alongside social media strategies, simple digital messaging via SMS and WhatsApp continues to be important, particularly in resource-constrained settings. A quasi-experimental study in Hong Kong found that SMS reminders significantly improved adherence to testing schedules and increased HIVST uptake in the intervention group ^28^. Additional evidence from a systematic review in LMICs shows that SMS campaigns can extend the reach of HIVST interventions and are relatively easy to integrate into health systems with limited resources ^39^. Their simplicity, low cost, and personal nature make SMS a reliable option for supporting reminder systems among key populations. WhatsApp also emerges as a promising medium for large-scale digital campaigns. The CHALO! 2.0 protocol in India highlights that WhatsApp Business can combine structured broadcasts, educational materials, and digital links with peer outreach, while maintaining user confidentiality ^35^. Given its popularity across several middle-income countries, WhatsApp enables personal, two-way communication that complements the role of SMS in expanding HIV testing uptake.

### Theme 4: Peer and network-based digital strategies

Beyond apps and short messaging, approaches that leverage social networks and peer support are increasingly prominent. These strategies harness interpersonal relationships to expand the reach of HIV testing. Evidence from the United States shows that peer-recruiter distribution of HIVST kits, facilitated by digital channels, reached people who rarely or never test and yielded higher positivity rates than conventional programs ^27^. Other mechanisms, including digital peer recruitment and referral links, have also broadened distribution and increased community participation. For example, unique referral links combined with monetary or digital coupon incentives produced significant gains in HIVST uptake ^35^ ^,^ ^37^. Peer-moderated online groups provide supportive social spaces for sharing experiences, offering emotional support, and reinforcing testing motivation. Studies from China and multi-country syntheses indicate that such groups can increase participation in testing ^39^ ^,^ ^40^. In conjunction, kit distribution, digital recruitment, incentive systems, and online peer support show that peer-based strategies are relevant and effective for increasing HIV testing uptake among key populations.

### Overall synthesis

Across the four themes, digital health promotion strategies appear complementary. Mobile apps offer integrated, personalized services; social media and web platforms broaden audience reach and convert interest into action; SMS and WhatsApp provide simple and affordable communication; and peer- and network-based strategies reinforce community engagement and the sustainability of testing behaviors. Used in combination, these approaches can address structural and social barriers while opening avenues for more contextual, scalable, and durable innovations to promote HIV testing among key populations.

## Discussion

This scoping review highlights the diverse and complementary roles of digital health strategies in promoting HIV testing uptake among key populations. Across the included studies, app-based and social media interventions were most frequently implemented among MSM and young MSM populations, while messaging and peer-network approaches were often used to boost engagement, outreach, and HIV self-testing support across community settings. Collectively, the findings suggest that digital HIV testing promotion functions as a multidimensional ecosystem rather than a single intervention pathway, with each strategy addressing distinct structural, psychosocial, and access-related barriers to HIV testing.

Mobile applications consistently emerged as a central digital innovation supporting HIV testing among key populations. Their primary advantage lies in providing flexible access to services, including ordering HIVST kits, receiving testing reminders, and accessing private digital counselling ^33^ ^,^ ^36^. Evidence from South Africa underscores the potential of mHealth to mitigate barriers related to distance, time, and stigma, thereby driving user engagement in HIV testing ^43^. Beyond access, apps build awareness and motivation through personalized reminders, integration with clinic-based services, and gamification elements ^29^ ^,^ ^36^. Other literature notes that digital health apps can drive behavior change by improving awareness, perceived usefulness, and user satisfaction ^34^ ^,^ ^44^. Behavioral models further suggest that perceived ease of use is positively associated with intention to undergo HIV testing ^44^ ^,^ ^45^. These findings are consistent with trial data from the United States showing that MyChoices and LYNX increased HIV testing uptake to 74% compared with standard care ^31^. Collectively, mobile apps function not only as promotional tools but also as comprehensive platforms that integrate education, motivation, and linkage to care for populations at elevated risk.

While apps emphasize individualized support, social media and web platforms offer wide reach to engage key populations. Studies show that social media campaigns can recruit MSM, sex workers, and sexual minority groups with high participation, sometimes outperforming volunteer-based strategies ^38^ ^,^ ^46^. Effectiveness is enhanced when content is culturally tailored, such as in bilingual campaigns for Latinx communities in the United States ^42^ ^,^ ^47^. Educational internet-based and peer-delivered digital interventions have been shown to address psychosocial and motivational barriers and to increase intentions and uptake of HIV testing among key populations in diverse settings ^48^. Targeted digital advertising has been reported as cost-effective in directing users to testing services, although ethical considerations remain notable ^32^ ^,^ ^49^. Web-based facilitation further improves access to HIVST through kit-ordering pages, live-chat support, and follow-up assistance ^39^ ^,^ ^40^. Considered in conjunction, social media and the web do not merely amplify message reach; they can convert interest into action by addressing structural, cultural, and psychosocial barriers.

In combination with broad-reach strategies, simpler digital interventions such as SMS and WhatsApp remain important, particularly in resource-constrained settings. SMS reminders improved adherence to HIVST in Hong Kong ^28^, and a systematic review indicates that SMS campaigns are relatively easy to integrate into health systems at low cost ^39^. Additional evidence suggests high acceptability and enhanced connectedness with health services, although effects on ART and PrEP adherence vary, this broader body of evidence highlights that digital health interventions extend beyond HIV testing and also play a role in supporting adherence and retention in HIV care ^50^ ^,^ ^51^ ^,^ ^52^. WhatsApp provides more interactive two-way communication. The CHALO! 2.0 protocol in India demonstrates the use of WhatsApp Business to combine multimedia messaging, educational materials, and peer outreach ^35^. Evidence from South Africa indicates the feasibility of WhatsApp-based microlearning among health workers ^53^. Studies in Peru and the United Kingdom also report benefits for peer support and higher engagement through online groups ^54^ ^,^ ^55^. Given their low cost, accessibility, and personal nature, digital messaging strategies show considerable promise, despite limitations such as the relatively low interactivity of SMS, dependence on digital literacy, and privacy concerns, which may also affect access among vulnerable populations even within high-income settings ^56^ ^,^ ^57^.

Beyond apps and short messaging, approaches that mobilize social networks and peer support are increasingly prominent. These strategies leverage interpersonal ties to distribute HIVST kits, share referral links, and provide emotional support online. Randomized evidence shows that peer-led online communities can increase acceptance of HIVST among African American and Latinx MSM ^58^, while a global meta-analysis reported 1.5-fold higher testing uptake among populations exposed to peer interventions compared with those unexposed ^41^. Network-based kit distribution has reached more first-time testers and provided higher positivity rates than conventional methods, with indications of higher cost-effectiveness ratios ^27^ ^,^ ^59^. The added value results from social support through motivation, positive experiences, and sustained engagement in online communities ^40^ ^,^ ^60^ ^,^ ^61^. Compared with apps, SMS, or generic social media, peer and network approaches foster a stronger sense of community ownership, although they face challenges such as the need for sustained peer commitment, risks of misinformation, and data security concerns ^62^. As such, these strategies are a critical complement to other digital approaches, especially for reaching high-risk groups who are difficult to engage through conventional channels; however, their effectiveness and scalability may vary across regions and health system contexts depending on differences in digital infrastructure, service integration, and the role of community-based and public health systems.

Despite this review providing a comprehensive overview of digital strategies for HIV testing among key populations, several limitations should be considered. First, heterogeneity across studies in design, target populations, and outcomes limits direct comparison and generalization. Second, much of the available evidence derives from small studies, pilot trials, or protocols, which constrains the strength of conclusions and underscores the need for more robust designs. Third, geographical coverage is dominated by middle- and high-income countries, with limited representation from low-income settings, where social contexts and digital infrastructure differ markedly. Fourth, details for intervention design, including intensity, duration, and specific digital components, are not consistently reported, which complicates replication and further evaluation. Fifth, representation remains limited for certain key populations, particularly transgender women and PWID, while broader vulnerable populations, such as cisgender women and girls, were outside the predefined scope of this review. Future research should prioritize digital HIV testing interventions tailored to underrepresented populations and explore how intervention effectiveness may differ across social, cultural, structural, and health system contexts.

## Conclusion

This scoping review mapped evidence on digital health promotion strategies designed to increase HIV testing among key populations. We identified four core themes, namely mobile applications, social media and web-based campaigns, digital messaging via SMS and WhatsApp, and peer and network-based strategies. In conjunction, these approaches constitute a complementary ecosystem, comprising from personalized service delivery to community support. The review underscores the strategic role of digital innovation in expanding access, reducing structural and psychosocial barriers, and increasing engagement in HIV testing among populations at elevated risk. From a medical practice and public health policy perspective, integrating multiple digital strategies may provide a more effective and sustainable approach, particularly in resource-constrained settings. Policymakers and healthcare professionals should consider culturally responsive and community-engaged digital interventions to improve equitable access to HIV testing among underserved populations. However, studies with more robust designs, broader geographic representation, and longer follow-up are needed to confirm effectiveness and sustainability.

## Data Availability

The sources of information used in the study are indicated in the body of the article.
