# Supplementary material for: Digital health promotion strategies to increase HIV testing uptake among key populations: a scoping review
Source: Cad Saude Publica. 2026 Aug 10;42:e00274825. doi: 10.1590/0102-311XEN274825 (PMC13460900; doi:10.1590/0102-311XEN274825)
Supplement: Supplementary file 1 [file 1678-4464-csp-42-EN274825-s.pdf]

Supplementary File 1. Database Search Strategies

Database	Search Strategy
**PubMed**	(("digital health"[MeSH Terms] OR "digital health"[Title/Abstract] OR "e-health"[Title/Abstract] OR "mHealth"[Title/Abstract] OR "telehealth"[Title/Abstract] OR "mobile application"[Title/Abstract]) AND ("HIV"[MeSH Terms] OR "HIV testing"[MeSH Terms] OR "HIV"[Title/Abstract] OR "HIV testing"[Title/Abstract]) AND ("promotion"[Title/Abstract] OR "strategy"[Title/Abstract] OR "campaign"[Title/Abstract] OR "intervention"[Title/Abstract]) AND ("MSM"[Title/Abstract] OR "men who have sex with men"[MeSH Terms] OR "men who have sex with men"[Title/Abstract] OR "transgender persons"[MeSH Terms] OR "transgender"[Title/Abstract] OR "trans women"[Title/Abstract] OR "sex workers"[MeSH Terms] OR "sex worker"[Title/Abstract] OR "female sex workers"[Title/Abstract] OR "FSW"[Title/Abstract] OR "people who inject drugs"[MeSH Terms] OR "PWID"[Title/Abstract] OR "key populations"[Title/Abstract] OR "adolescents"[MeSH Terms] OR "youth at risk"[Title/Abstract]))
**Scopus**	(TITLE-ABS-KEY("digital health" OR "e-health" OR mHealth OR telehealth OR "mobile application") AND TITLE-ABS-KEY("HIV" OR "HIV testing") AND TITLE-ABS-KEY("promotion" OR "strategy" OR "campaign" OR "intervention") AND TITLE-ABS-KEY("MSM" OR "men who have sex with men" OR transgender OR "trans women" OR "sex workers" OR "female sex workers" OR FSW OR PWID OR "people who inject drugs" OR "key populations" OR adolescents OR "youth at risk"))
**Cochrane Library**	(("digital health" OR "e-health" OR mHealth OR telehealth OR "mobile application"):ti,ab,kw AND ("HIV" OR "HIV testing"):ti,ab,kw AND ("promotion" OR "strategy" OR "campaign" OR "intervention"):ti,ab,kw AND ("MSM" OR "men who have sex with men" OR transgender OR "trans women" OR "sex workers" OR "female sex workers" OR FSW OR PWID OR "people who inject drugs" OR "key populations" OR adolescents OR "youth at risk"):ti,ab,kw)
**CINAHL**	((MH "Digital Health" OR TX "digital health" OR TX "e-health" OR TX mHealth OR TX telehealth OR TX "mobile application") AND (MH "HIV" OR MH "HIV Infections" OR MH "HIV Testing" OR TX "HIV" OR TX "HIV testing") AND (TX "promotion" OR TX "strategy" OR TX "campaign" OR TX "intervention") AND (MH "Men who have sex with men" OR TX MSM OR TX "men who have sex with men" OR MH "Transgender Persons" OR TX transgender OR TX "trans women" OR MH "Sex Workers" OR TX "sex workers" OR TX "female sex workers" OR TX FSW OR MH "Substance Abuse, Intravenous" OR TX "people who inject drugs" OR TX PWID OR TX "key populations" OR MH Adolescents OR TX adolescents OR TX "youth at risk"))

Supplementary File 2. Data Extraction Form

Variable	Description
Author(s), year	Study identification (first author and publication year)
Country/region	Location where the study was conducted
Population	Target population (e.g., MSM, transgender people, sex workers, PWID, adolescents at increased risk)
Sample size	Number of participants included in the study
Study design	Type of study (e.g., randomized controlled trial, quasi-experimental, cross-sectional, mixed-methods, protocol)
Study objective	Main aim or purpose of the study
Digital health strategy/intervention	Type of digital health promotion strategy or intervention used
Outcome (HIV testing uptake)	Key findings related to HIV testing uptake or engagement
Thematic category/subtheme	Assigned theme or subtheme based on thematic analysis
Notes	Additional relevant information (e.g., implementation context, limitations, or remarks)
